# Efficient and Sensitive Electrically Small Rectenna for Ultra-Low Power RF Energy Harvesting

**DOI:** 10.1038/s41598-018-33388-w

**Published:** 2018-10-09

**Authors:** Stylianos D. Assimonis, Vincent Fusco, Apostolos Georgiadis, Theodoros Samaras

**Affiliations:** 1 School of Electronics, Electrical Engineering and Computer Science, Queen’s University Belfast, Belfast, BT3 9DT United Kingdom; 2 School of Engineering and Physical Sciences, Heriot-Watt University, Edinburgh, EH14 4AS United Kingdom; 3 School of Physics, Aristotle University of Thessaloniki, Thessaloniki, 54124 Greece

## Abstract

A new electrically small antenna with size *ka* = 0.415 is presented, fabricated and measured in this work. This is intrinsically matched to 50 Ω, has omni-directional and linear-polarized radiation pattern in the horizontal plane with maximum directivity of 1.75 dBi and simulated radiation efficiency of 93%. The antenna in combination with a low-complex and co-planar rectifier with one single diode forms a high efficient and sensitive electrically small rectenna with *ka* = 0.53 at 868 MHz (UHF RFID-band in Europe). The latter has measured efficiency 22.5% for −19 dBm power input and sensitivity of −44 dBm (or equivalently 0.00028 *μ*W/cm^2^ power density), while at 2.25 *μ*W/cm^2^ is able to supply continuously, i.e., without a boost converter or use of any energy tank, a small electrical device with 118 *μ*W. In order to increase the dc output voltage and the delivered dc power to the load for lower power density levels, rectenna-array configuration is exploited. Application to batteryless, backscatter wireless sensor node powering is discussed. Specifically, for a power density of 0.1237 *μ*W/cm^2^ the RF energy harvesting system delivers 172 *μ*W at 2.85 V every 22.5 s.

## Introduction

The development of new deployed wireless mobile technologies such as Multi-Input Multi-Output (MIMO) systems, Wireless Local Area Networks (WLAN), Radio Frequency Identification (RFID) and Wireless Sensor Networks (WSN) leads to the need for antennas with relatively small size, but without antenna performance degradation. Based on the latter, electrically small antennas have gained increasing attention in recent years^1–9^ since Wheeler’s study^10^.

There are many definitions for the electrically small antennas: based on Wheeler’s criterion an antenna is electrically small when it can be circumscribed by a radian sphere of radius $\lambda /2\pi$^10^, while based on King’s criterion^11^, the accepted electrical size limit for an electrically small antenna is $ka\le 0.5$, where $k=2\pi /\lambda$ is the wavenumber and *a* is the radius of a sphere, which encloses the antenna.

Electrically small antenna characteristics consist of relatively small size (i.e., a small fraction of the wavelength), low input resistance (i.e., highly capacitive/inductive input impedance), and characteristic omnidirectional radiation pattern. Usually, small antenna impedance matching to 50 ${\rm{\Omega }}$ load is a difficult procedure, while the radiation pattern tends to approach the typical dipole omnidirectional radiation pattern, with maximum directivity equal to 1.5^12^.

Energy harvesting through RF-to-dc rectification^13^, has been gaining ground over the last decade^7–9,14–27^. The number of RF emitters has been rapidly increasing due to the development of new wireless technologies and it remains an engineering challenge how to capture unused ambient RF energy and use this to supply small electrical devices, such as backscatter radio sensor networks^28–30^.

In typical RF-to-dc rectification systems, an antenna is combined with a rectifier, which mainly consists of one or more diodes in specific configurations, forming a *rectenna*. The main design limitation in a rectenna is the relatively low available ambient power density level as well as the *sensitivity* (i.e., the ability to harvest energy and operate at low power density). Based on long-term RF EMF measurements in the European Union (EU)^31^, power density varies from 0.0017 to 0.8594 *μ*W/cm^2^. Hence, it is clear that, in order to fully exploit and harvest the RF energy available in the environment, rectennas should be designed to operate at ultra-low power densities. In order to increase the sensitivity for a given power density, losses inserted by the rectifier diodes, the matching network and the dielectric losses in the substrate should be limited. Finally, since the location of the source is not a-priori known, the rectenna should have an omni-directional radiation pattern.

The use of electrically small antennas in rectennas^7–9^, has two main advantages: a) a matching network between the antenna and the rectifier can be avoided due to the intrinsic highly capacitive/inductive input impedance of these antennas, and b) the rectennas have relatively compact size.

The goals and the contribution of this work are not only 1) to design an electrically small antenna, which 2) is intrinsically matched to 50 ${\rm{\Omega }}$, 3) has omni-directional radiation pattern in the horizontal plane and 4) has high radiation efficiency (RE), but also, 5) to design an electrically small rectenna, which has 6) high RF-to-dc efficiency for low power input (i.e., less than −10 dBm), 7) high sensitivity (i.e., equal or above 0.0017 *μ*W/cm^2^), and 8) omni-directional radiation pattern appropriate for ambient energy harvesting.

Based to our knowledge and according to the relevant literature (an overview is given in Tables 1 and 2), the presented electrically small rectenna has better performance in terms of efficiency for low power input and sensitivity compared to prior-art designs. It is noted that the comparison, in order to be fair, relates to only electrically small antennas and rectennas.

**Table 1:** Electrically Small Antenna Comparisons.

	*ka*	gain (dBi)	RE (%)	radiation pattern
^2^	0.645	2.03^+^	71.6^+^	directional
^3^	0.498	0.53^+^	75^+^	nearly isotropic
^4^	0.497	3.69*	81.88*	directional
^5^	0.46	1.77*	90.86*	nearly omni-directional
^6^	0.45	1.99*	96.8*	omni-directional
^7^	0.467	1.063*	66.5*	omni-directional
^8^	0.996	1.87*	–	omni-directional
^9^	0.629	1.33*	92.3*	directional
this work	0.415	1.43*	93*	omni-directional

^+^Measured, *Simulated.

**Table 2:** Electrically Small Rectenna Comparisons: rectennas have the same radiation pattern with the relevant antennas (Table 1).

	*ka*	P_in_ (dBm)	*η* (%)	matching network
^7^	0.611	0	79.6^+^	no
^8^	0.996	−9	37^+^	yes
^9^	0.604	0	67.9^+^	no
this work	0.53	−19	22.5^+^	no

^+^Measured.

## Results

The small antenna is first presented in terms of geometry, performance (i.e., reflection coefficient and radiation pattern), antenna radiation mechanism and parametric analysis. Next, the coplanar rectifier is presented and its input impedance and RF-to-dc efficiency is estimated via simulation and measurement. The RF-to-dc and the dc output voltage of the electrically small rectenna versus power input and power density is then presented, while finally backscatter, wireless sensor node powering measurements take place.

### Small Antennas

#### Antenna Design

**Figure 1:**
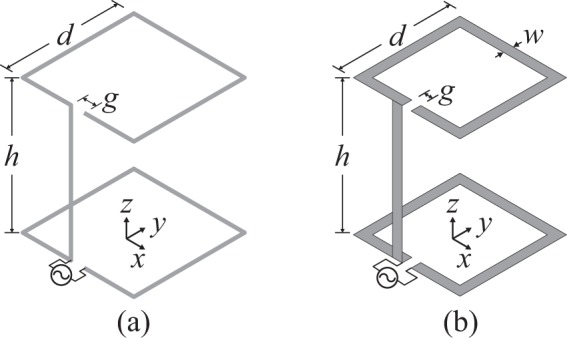
Proposed antenna geometry: (**a**) wire-SSRR with $d=24.3634$ mm ($\lambda /14.2$), $h=29.8597$ mm ($\lambda /11.6$), $g=2.4333$ mm, $r=0.2$ mm and (**b**) strip-SSRR with $d=26.446$ mm ($\lambda /13.1$), $h=33.8207$ mm ($\lambda /10.2$), $g=2.2566$ mm, $w=2.43$ mm.

The proposed antenna consists of two electrically small square split ring resonators (SSRRs) with maximum edge dimension *d* and gap *g*, which are located at distance *h*. The two SSRRs are electrically connected, while antenna feeding was applied across the gap of one SSRR. Two types of these antennas where fabricated: the first (“wire-SSRR” antenna) is made of wire with radius *r* (Fig. 1a), while the second (“strip-SSRR” antenna) is made of strip line with width *w* (Fig. 1b).

The antennas were designed in order to operate (i.e., reflection coefficient relative to 50 ${\rm{\Omega }}$ less than −10 dB) at 868 MHz, taking into account the allocated UHF RFID frequencies in Europe^32^. It is noted that in our simulations, an SMA connector with maximum dimension (i.e., height) of 5 mm was placed and taken into account. The latter was crucial for the design procedure because a) in our measurements an SMA was used for feeding, b) the connector’s dimensions are relatively large compared with the dimensions of the proposed antennas, and c) it was found that the SMA presence influences the antenna performance in terms of both reflection coefficient and radiation pattern. In the strip-SSRR case, foam material was used for mechanical support with ${\varepsilon }_{r}=1.0001$. After optimization with the Quasi Newton algorithm (Ansys HFSS), the optimal obtained dimensions for both antennas are given in the caption of Fig. 1: for the wire-SSRR $ka=0.415$, while for the strip-SSRR $ka=0.459$, and thus both meet King’s criterion^11^ ($ka\le 0.5$).

**Figure 2:**
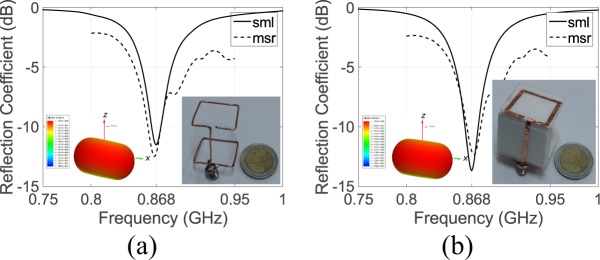
Wire-SSRR (**a**) and strip-SSRR (**b**) antenna simulated (sml) and measured (msr) reflection coefficient.

The proposed antennas simulated reflection coefficient is depicted in Fig. 2. Both antennas operate at 868 MHz. Specifically, the wire- and strip-SSRR antenna has a frequency impedance bandwidth relative to 50 ${\rm{\Omega }}$ of 1.56% (i.e., 859.5–873 MHz) and 1.64% (i.e., 863–877.3 MHz), respectively. Figure 2 also depicts the measured reflection coefficient, where good agreement is observed, and the simulated 3D directivity as inset for the both antennas.

It is noted that the reflection coefficient estimation was based only on full-electromagnetic analysis. Estimation through the equivalent circuit model e.g.^33–36^, could be used, as well. However, the full-electromagnetic study was adopted because, in general, is more accurate since takes into account the real shape of the geometry, the total coupling between the antenna parts and the antenna radiation.

**Figure 3:**
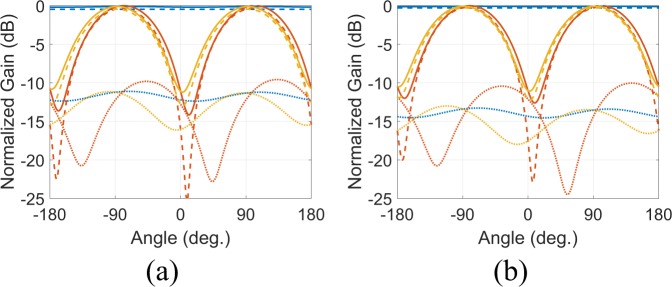
The total (solid lines), co-polar (dashed lines) and cross-polar (dotted lines) simulated normalized gain for the wire-SSRR (**a**) and the strip-SSRR (**b**) antenna in the *xy*– (blue lines) *xz*– (red lines) and *yz*–plane (yellow lines).

The total simulated normalized gain (solid lines) of the proposed antennas is depicted in Fig. 3. The wire- and strip-SSRR antennas both have maximum half-power beamwidth of 96° in the vertical plane (i.e., *xz*-plane). They also have omnidirectional radiation pattern with the maximum gain occurring in the *xz*-plane at $\theta =-\,78^\circ ,102^\circ$ (wire-SSRR) and at $\theta =-\,80^\circ ,100^\circ$ (strip-SSRR). The maximum simulated directivity was 1.75 and 1.7 dB, for the wire- and strip-SSRR antenna, respectively while the simulated gain is 1.43 (wire-SSRR) and 1.38 dB (strip-SSRR), resulting in a simulated radiation efficiency of 93% for both antenna types. In Fig. 3 is also depicted the simulated co- (dashed lines) and cross-polar (dotted lines) normalized gain of the proposed antennas: in the *xy*-plane (blue lines) and for both the antennas, the cross-polar component, i.e., component which lies in the *xy*-plane (dotted lines) is always lower than −10 dB, which reveals that the antennas in the horizontal plane are linear vertical polarized.

Hence, based on Table 1, the proposed design and especially the wire-SSRR, is the smallest antenna with the highest RE and simultaneously has omni-directional radiation pattern in the horizontal plane, reported to date. These characteristics make it appropriate for RF energy harvesting and RFID applications.

#### Radiation Mechanism

**Figure 4:**
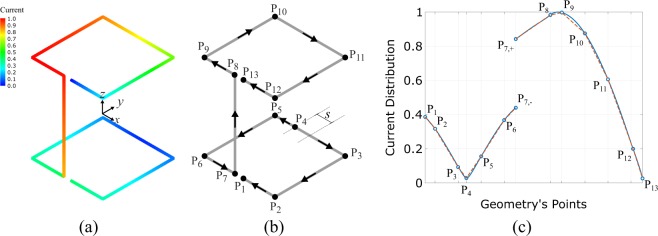
The simulated normalized magnitude (**a**) and the direction/vectors (**b**) of the wire-SSRR antenna’s current distribution. The normalized magnitude of the current distribution along the geometry’s points: the continuous line represents the numerically estimated values, while the dashed line depicts the linear approximation, which will be used through the analytical solution of the electrical component in the far-field region (**c**).

The normalized magnitude and the direction (vectors) of the wire-SSRR antenna’s current distribution is depicted in Fig. 4a,b, respectively. Figure 4c depicts the normalized magnitude of the current distribution along the geometry’s points. It is observed that currents flow in-phase in the vertical and upper loop, while in the bottom loop there are two separated regions: the currents flow in-phase from *P*_4_ to *P*_1_ and from *P*_4_ to *P*_7_, respectively. The maximum current distribution is located in the vertical wire, as well as in part of the upper loop, and thus, power is radiated mainly through these parts, resulting vertical polarization, as mentioned. The bottom loop, where the feed was applied, radiates less and operates as a magnetic resonator, which delivers power to the rest of the structure; at point *P*_7_ (Fig. 4b) current is added and flows upwards, towards *P*_8_ and for this reason there is a discontinuity in the current distribution, i.e., current magnitude at *P*_7,−_ (from *P*_6_ to *P*_7_) differs from that at *P*_7,+_ (from *P*_7_ to *P*_8_), as depicted in Fig. 4c.

In order to analytically estimate the electrical field of the proposed antenna in the far-field region and to study in-depth the antenna radiation mechanism, it is assumed that the antenna consists of twelve de-coupled, thin wires, each of these located between points *P*_j_*P*_j+1_, where $j=1,2,\ldots ,13$, as it is depicted in Fig. 4b, with linear distribution, which in the $i$-th edge is given by,

1 $${{\bf{J}}}_{i}={J}_{i}({z}_{i}^{\prime} ){\hat{z}}_{i}=\{\frac{{I}_{u}-{I}_{l}}{{L}_{i}}({z}_{i}^{\prime} -\frac{{L}_{i}}{2})+{I}_{u}\}{\hat{z}}_{i},$$

where $I({P}_{u})$, $I({P}_{l})$ is the current value at the upper, lower point of the *i*-th edge with $i=1,2,\ldots ,12$, respectively, aligned with the current direction, while the *L*_*i*_ is the edge length. Hence, the equivalent current distribution of the antenna is depicted in Fig. 4c with the dashed line.

The electrical field of a finite dipole antenna, which lies in the *z*_*i*_-axis, which is in parallel with the *i*-th edge, has length *L*_*i*_, ideally zero thickness and current distribution ${{\bf{J}}}_{i}={J}_{i}\,({z}_{i}^{^{\prime} })\,{\hat{z}}_{i}$ is given by,

2 $${{\bf{E}}}_{i}={E}_{{\theta }_{i}}({r}_{i},{\theta }_{i},{\varphi }_{i}){\hat{\theta }}_{i}$$

in far-field region, where,

3 $${E}_{{\theta }_{i}}({r}_{i},{\theta }_{i},{\varphi }_{i})=j\frac{\eta k}{4\pi }\frac{{e}^{-jk{r}_{i}}}{{r}_{i}}\,\sin \,{\theta }_{i}\,{\int }_{-{L}_{i}/2}^{{L}_{i}/2}\,{J}_{i}({z}_{i}^{\prime} ){e}^{jk{z}_{i}^{\prime} \cos {\theta }_{i}}\,d{z}_{i}^{\prime} ,$$

where $k=2\pi /\lambda$ and $\eta \approx 120\,\pi$ is the free-space wave-number and impedance, respectively, and the integral represents the antenna’s *space factor*^33^. After calculation, Eq. (2) via Eq. (3) is written as,

4 $${{\bf{E}}}_{i}=\tfrac{\eta }{4\pi k{L}_{i}}\tfrac{{e}^{-jkr}}{r}\,\tan \,{\theta }_{i}\{k{L}_{i}({I}_{u}-{I}_{l})\,\cos \,\tfrac{k{L}_{i}\,\cos \,{\theta }_{i}}{2}+[\tfrac{2}{\cos \,{\theta }_{i}}({I}_{u}-{I}_{l})+jk{L}_{i}({I}_{u}+{I}_{l})]\,\sin \,\tfrac{k{L}_{i}\,\cos \,{\theta }_{i}}{2}\}.$$

Hence, the total electrical field is given by^33^,

5 $${\bf{E}}=\sum _{n=1}^{12}\,{{\bf{E}}}_{i}.$$

**Figure 5:**
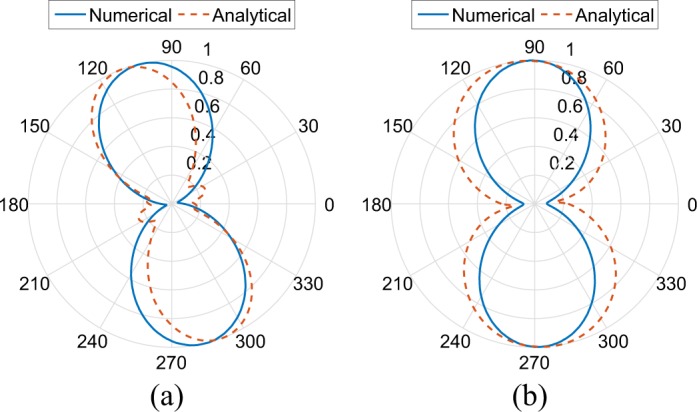
Wire-SSRR directivity in the *xz* (**a**) and *yz* (**b**) -plane as it is estimated via the analytical and the numerical procedure.

Figure 5 depicts the magnitude of the electrical component in the far-field region, as estimated via Eq. (5). It shows agreement between the two methods (i.e., numerical and analytical), while the shift in angle and the beam-width variation is the result of the following assumptions: in the analytical solution a) the inter-edge mutual coupling was not taken into consideration, b) the radius of the wire was considered as zero (0.2 mm in practice) c) linear variation of the current distribution along the edges of the antenna is used (Fig. 4c). It is noted that the analytical study is presented a) to study in-depth the antenna radiation mechanism and b) to obtain an analytical formula, which depends only on the design parameters.

#### Parametric Analysis

**Figure 6:**
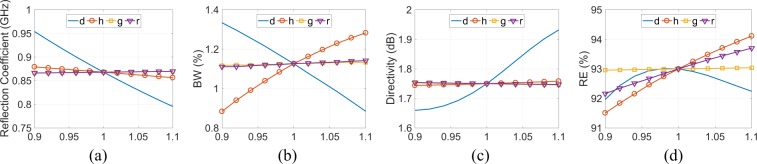
Simulated wire-SSRR RE (**a**) and impedance BW relative to 50 ${\rm{\Omega }}$ (**b**) versus design parameters *d*, *h*, *g*, and *r*: the variation of each parameter is from 90% to 110% of each initial value.

Figure 6 depicts the frequency at minimum reflection coefficient, the bandwidth (BW) relative to $50\,{\rm{\Omega }}$, the maximum directivity at 868 MHz and the RE (%) of the wire-SSRR versus all the design parameters: the variation of each parameter is from 90% to 110% of each initial value. In all the latter cases, the antenna resonates, i.e., the reflection coefficient is below −10 dB. It is evident that the resonance frequency is mostly affected by the *d* parameter, e.g., 110% increment of *d* shifts the resonance frequency to 800 MHz. BW is affected by both *d* and *h* in an opposite way: as the *d* and *h* increase, the BW decreases and increases, respectively. Directivity is again mostly affected by parameter *d* and increases with the *d* increment. RE is affected by all the parameters, except for the gap *g*. Specifically, RE increases as *h* and *r* increase, however, there is an optimum value for the *d* parameter. Thus, it is possible to enhance the BW and the RE by increasing the parameter *h*, i.e., the height of the antenna, without affecting too much the operating frequency and the maximum directivity.

### Coplanar Rectifier

**Figure 7:**
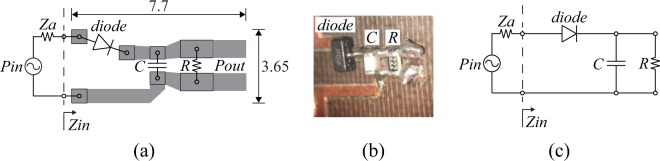
Coplanar rectifier topology, where there is no ground plane (**a**), picture of the fabricated geometry (zoomed by microscope) (**b**) and equivalent circuit schematic (single-series circuit with a single diode performing half-wave rectification) (**c**). Also, depicted the dimensions of the design in mm, while the footprint of the diode, the capacitor and the resistor is SOT-323, 0402 and 0603, respectively.

A rectifier was designed to operate at 868 MHz for a low-power input of −20 dBm. Its geometry is coplanar (i.e., there is no ground plane) and it was chosen in order to facilitate easy connection with the antennas SSRR gap. For mechanical stability and fabrication ease, Taconic TLY-5 substrate (${\varepsilon }_{r}=2.2$, $\tan \,\delta =0.0009$) with thickness of 0.51 mm was used. The design, the zoomed fabricated geometry and the equivalent circuit schematic are depicted in Fig. 7: a single series circuit, which utilizes one low-cost Schottky diode “HSMS285B” (Avago Technologies, Inc.), one capacitor and a load, performs half-wave RF-to-dc rectification. There is no matching network since the antenna will be directly impedance matched to the rectifier at a next step. The rectifier’s input impedance is a function of the power input, the operating frequency and the rectifier’s output load, and thus, the goal of the simulation was to estimate the rectifier’s input impedance and output load when the RF-to-dc efficiency (i.e., the ratio of the dc power output to the RF power input) is maximized for power input of −20 dBm at 868 MHz: the maximum RF-to-dc efficiency came to 20% with rectifier’s input impedance of ${{\rm{Z}}}_{{\rm{r}}}=135-j507\,{\rm{\Omega }}$ and optimum output load of $2193$ ${\rm{\Omega }}$.

**Figure 8:**
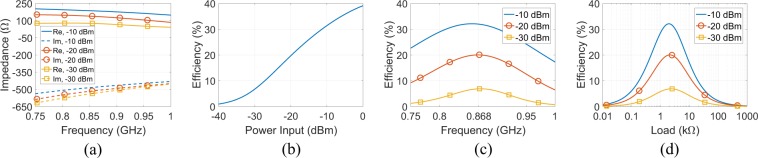
Rectifier’s input impedance (real (Re) and imaginary (Im) part) versus frequency for various levels of power input (**a**), rectifier’s RF-to-dc efficiency versus power input at 868 MHz (**b**), versus frequency (**c**) and versus load at 868 MHz (**d**) for various levels of power input. At (**a**–**c**) the output load was fixed at 2193 ${\rm{\Omega }}$, while at (**b**–**d**) source was impedance matched to the rectifier (i.e., ${Z}_{{\rm{s}}}=135+j507$). All the results are based on simulations.

Figure 8a depicts the simulated input impedance in terms of real (Re) and imaginary (Im) part versus frequency for various power input levels: it is evident that the rectifier is capacitive and non-linear, while at 868 MHz the Re and Im part of the input impedance varies from 74 to 184 ${\rm{\Omega }}$ and from −528.5 to −477.6 ${\rm{\Omega }}$, respectively, from −30 to −10 dBm power input, respectively. Next, the input impedance of the generator was fixed at ${{\rm{Z}}}_{{\rm{s}}}=135+j507\,{\rm{\Omega }}$, in order to be matched with the rectifier, and the RF-to-dc efficiency versus power input at 868 MHz was estimated. Figure 8b shows the simulated results: the efficiency increases from 1% to 39.12% for power input from −40 to 0 dBm, while for −20 dBm is 20%. Figure 8c,d depicts the RF-to-dc efficiency versus frequency (load was fixed at 2193 ${\rm{\Omega }}$) and load (at 868 MHz), respectively, for various power input levels, when the rectifier is powered by an impedance matched source (i.e., again ${{\rm{Z}}}_{{\rm{s}}}=135+j507\,{\rm{\Omega }}$): higher power input results higher efficiency, as expected, but in slightly lower frequency (Fig. 8c) and load (Fig. 8d).

### Small Rectenna

The strip-SSRR antenna was redesigned a) in order to be impedance matched with the rectifier, and b) for direct connection to the latter, to form a rectenna. The obtained dimensions were: $d=19.21$ mm ($\lambda /18$), $h=52.06$ mm ($\lambda /6.6$), $g=2.45$ mm and $w=1.89$ mm (i.e., $ka=0.53$), while the antenna impedance was equal to ${{\rm{Z}}}_{{\rm{a}}}=127.3+j535.6\,{\rm{\Omega }}$ at 868 MHz, resulting in a reflection coefficient

6 $$|{\rm{\Gamma }}|=|\frac{{Z}_{{\rm{a}}}-{Z}_{{\rm{r}}}^{\ast }}{{Z}_{{\rm{a}}}+{Z}_{{\rm{r}}}}|$$

of −22.1 dB, where ${{\rm{Z}}}_{{\rm{r}}}=135-j507$ is the input impedance of the rectifier at 868 MHz and for −20 dBm power input, as mentioned. The antenna is slightly electrically larger than the initial strip-SSRR antenna with impedance of 50 ${\rm{\Omega }}$, (i.e., for the latter was $ka=0.459$) mainly because now the antenna impedance is different. Additionally, the antenna size is affected by the dielectric permittivity of the used substrate, which is now ${\varepsilon }_{r}=2.2$ (i.e., Taconic TLY-5), instead of ${\varepsilon }_{r}=1.0001$ (i.e., foam), which is used in the initial strip-SSRR antenna. However, the rectenna can be circumscribed by a radian sphere of radius $\lambda /2\pi$, and thus meets Wheeler’s criterion^10^.

**Figure 9:**
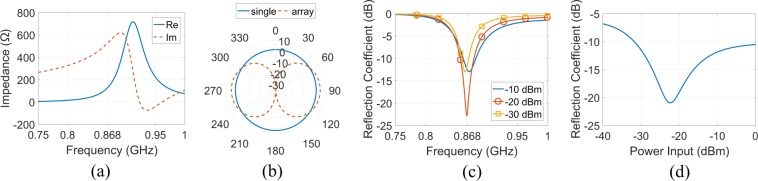
Antenna’s impedance in terms of Re and Im part versus frequency (**a**), gain in the horizontal plane (i.e., *xy*-plane) for a single antenna and an antenna-array of two elements placed side-by-side at distance *λ*/2 (b), rectenna’s reflection coefficient versus frequency for various levels of input power (**c**) and versus power input at 868 MHz (**d**). At (**c**,**d**) the rectenna’s load was 2193 ${\rm{\Omega }}$. All the results are based on simulations.

The antenna’s impedance in terms of Re and Im part versus frequency is depicted in Fig. 9a: the real part is maximized at 911 MHz, while the antenna is mainly inductive except the frequency region from about 923 to 961 MHz. In Fig. 9b the gain in the horizontal plane (i.e., *xy*-plane) is presented for a single antenna and an antenna-array of two elements in distance $\lambda /2$: in the first case the simulated gain is omnidirectional with maximum amplitude of 1.8 dB, while for the antenna-array the gain is directional with maximum of 4.8 dB, as expected based on theory^33^. Next, the antenna was connected to the rectifier, forming a rectenna and the reflection coefficient between these two parts was estimated. First, the power input was fixed and the reflection coefficient versus frequency is depicted in Fig. 9c: in all cases, antenna and rectifier are well impedance matched, while specifically for −30, −20 and −10 dBm the operating frequency bandwidth (i.e., $|{\rm{\Gamma }}| < -\,10$ dB) is 858–871, 856–879 and 861–882 MHz, respectively. Then, the frequency was fixed at 868 MHz and the reflection coefficient versus power input is presented in Fig. 9d: it is evident that the antenna is impedance matched with the rectifier at 868 MHz for power input from −31.8 to above 0 dBm, which makes the rectenna able to harvest RF energy from low up to higher power levels. It is noted that, in both cases, the rectenna’s output load was fixed at 2193 ${\rm{\Omega }}$.

**Figure 10:**
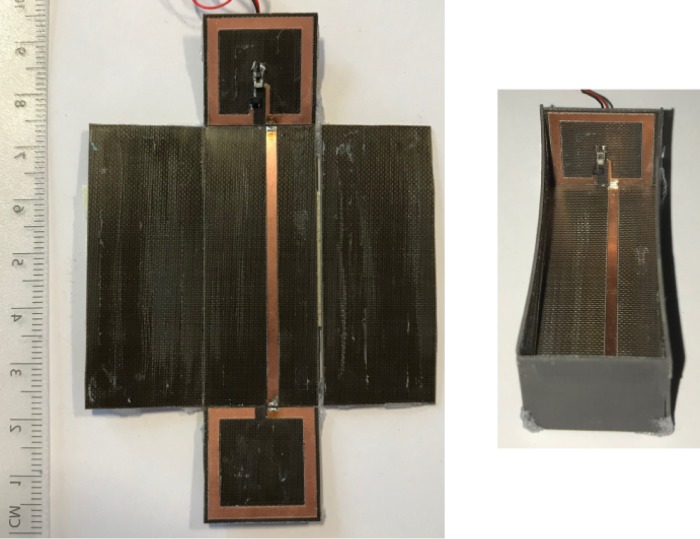
Fabricated and measured rectenna before (left) and after (right) is assembled in 3D shape: is also depicted the co-planar rectifier (upper SSRR).

**Figure 11:**
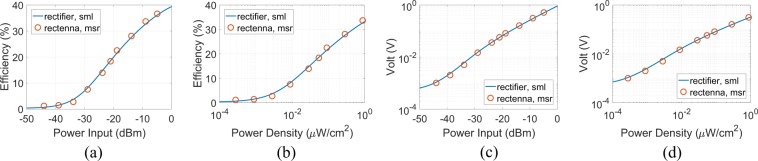
Simulated (sml) rectifier’s and measured (msr) rectenna’s RF-to-dc efficiency versus power input (**a**) and power density (**b**). Also depicted the simulated rectifier’s and measured (msr) rectenna’s dc output voltage versus power input (**c**) and power density (**d**). At all cases frequency was fixed at 868 MHz and load was 2193 ${\rm{\Omega }}$.

The antenna and the rectifier were fabricated forming the rectenna (Fig. 10). At 868 MHz the simulated (sml) rectifier’s efficiency and the measured (msr) rectenna’s efficiency versus power input and power density for output load of 2193 ${\rm{\Omega }}$ is depicted in Fig. 11a,b: for −19 dBm power input the measured RF-to-dc efficiency is 22.5%, while for the higher power input level of −14 dBm the efficiency is measured at 28%. Based on the latter results, it is evident that the rectenna presents superior performance in terms of RF-to-dc efficiency for low power input compared with other published electronically small rectennas (Table 2). The measured efficiency for the lowest power input of −44 dBm, where the rectenna operates, is 1.13%, and thus, the proposed harvester also presents high sensitivity (i.e., ability to harvest energy and operate at low power input or density) since based on the literature harvesting systems rarely operate lower than −30 dBm^22^. The maximum measured efficiency is 36.6% for −4.9 dBm power input. The simulated rectifier’s and the measured rectenna’s RF-to-dc efficiency at 868 MHz, but now versus power density is depicted in Fig. 11b: based on Eqs (10) and (11) (as it will be next explained in the measurement setup section), power input of −4.9, −14, −19 and −44 dBm corresponds to power density of 2.251, 0.277, 0.088 and 0.00028 *μ*W/cm^2^, respectively, and once again it is observed that the rectenna’s sensitivity is high since operates from 0.00028 *μ*W/cm^2^, which makes it a good candidate for RF energy harvesting for environments with ultra-low power density. The measured rectenna’s voltage output across the optimum load of 2193 ${\rm{\Omega }}$ versus power input and power density is depicted in Fig. 11c,d, respectively. For −4.9, −14 and −19 dBm power input, the voltage is 509.5, 157 and 79 mV, respectively, while for the power input of only −44 dBm the dc output voltage is 1 mV. For all the above cases, it is evident that there is a good agreement between measured and simulated results.

According to^31^, the values of (rms) electric field in the telecommunications spectrum of 10 MHz–6 GHz for 99% of outdoor measurements are below 1 V/m in EU. Specifically, based on long-term RF EMF measurements, the mean electric field strengths were between 0.08 V/m and 1.8 V/m, or equivalently power density was between 0.0017 and 0.8594 *μ*W/cm^2^, given that $S={E}_{{\rm{rms}}}^{2}/120\pi$. The latter indicates that the sensitivity of harvesters should be as low as 0.0017 *μ*W/cm^2^. Based on the simulated and measured results presented in Fig. 11, the efficiency of the rectenna varies from about 2.5% to 32.5% as the power density increases from 0.0017 to 0.8594 *μ*W/cm^2^. Additionally, in^37^ it was shown that the power density levels from a GSM-900 base station at a distance from 25 to 100 m varies from 0.01 to 0.3 *μ*W/cm^2^, and thus, again, the proposed rectifier covers the latter power density region.

### Supplying Backscatter Sensor Tags

In this section, the ability of the presented rectenna in supplying of small electrical devices, such as backscatter sensor tags, is discussed.

**Figure 12:**
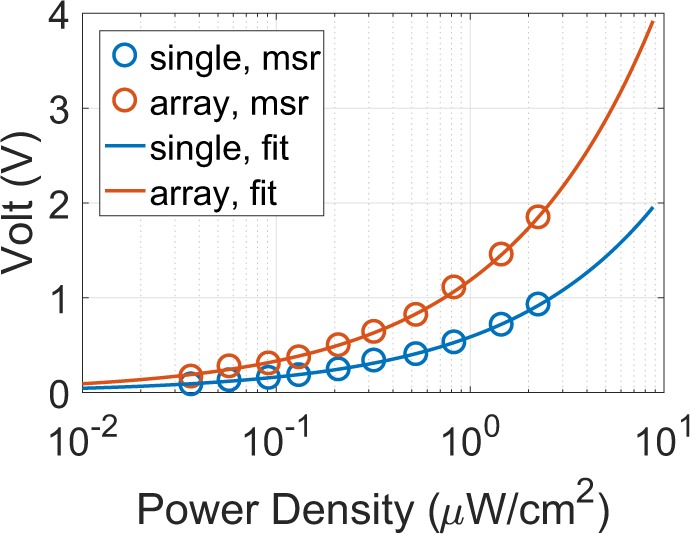
The measured, open-circuit dc output voltage versus power density of the single and the rectenna-array: two single rectennas were placed in the horizontal plane (i.e., side-by-side) at distance *λ*/2 and were also electronically connected in series configuration (i.e., voltage summing). The fitting curves for the both cases are also depicted: for the single rectenna is $V=0.5872\,{S}^{0.5542}$, while for the rectenna-array $V=1.185\,{S}^{0.5511}$, where *V* and *S* is in V and *μ*W/cm^2^, respectively.

The dc output voltage versus power density when the rectenna is opened-circuited was measured through a specific procedure, which will be next explained in the measurement setup section. Figure 12 depicts the results: as the measured power density varies from 0.04 to 2.25 *μ*W/cm^2^, the voltage varies from 98 to 925 mV. The fitting curve, which is also depicted, is given by $V={a}_{i}\,S{}^{{b}_{i}}$, with ${a}_{i}=0.5872,{b}_{i}=0.5542$ and *V* in V and *S* in *μ*W/cm^2^.

Next, in order to increase not only the open circuited dc output voltage, but also the total, delivered to the load dc power, two of the proposed rectennas were placed in the horizontal plane (i.e., side-by-side) at distance $\lambda /2$ and were also dc side connected, in series configuration (i.e., voltage summing). In the latter harvesting system, at the RF side each antenna element is terminated to a *hypothetical load*, which represents the input impedance of the co-planar rectifier, and hence, the harvester acts as a rectenna-array of two impedance matched antenna elements, despite the fact that the rectennas are connected after the rectification, at the dc side^20,22^. Equivalently, the two rectennas are loading each other at the near-field leading to mutual coupling which results directive radiation patter at the far-field. Consequently, the inter-element distance has impact to the far-field^33^ of the harvesting system, and thus, to the dc added power. The latter was the reason the inter-element distance of $\lambda /2$ was used. The radiation pattern of the rectenna-array is identical with the antenna-array pattern, depicted in Fig. 9b. The measured dc output voltage versus power density for the open-circuited rectenna-array is also depicted in Fig. 12. Now, the voltage is enhanced, compared to the single rectenna: for 0.04 and 2.25 *μ*W/cm^2^ the voltage is 201 mV and 1.85 V, respectively. The fitting curve for the dc open circuited voltage versus power density for the rectenna-array is now given by ${\rm{V}}=1.185\,{S}^{0.5511}$ and is depicted in Fig. 12, as well. All the above results are presented in Table 3, where is also included the RF power input *P*_in_ into the harvester as estimated by Eq. (13) when *G* equals 1.8 and 4.8 dB for the single and the rectenna-array, respectively and *G*_cal_ = 1.8 dB.

**Table 3:** RF Harvesting System Supplying Batteryless, Backscatter Wireless Sensor Node^29^.

RF Harvesting system	*S* (*μ*W/cm^2^)	*P*_in_ (dBm)	*V*_o.c._ (V)	Δ*τ*_*a*_ (s)	Δ*τ*_*b*_ (s)	Δ*τ*_*c*_ (s)	${{\boldsymbol{\eta }}}_{{\bf{t}}{\bf{o}}{\bf{t}},{\boldsymbol{\Delta }}{{\boldsymbol{\tau }}}_{{\boldsymbol{a}}}}$ (%)	${{\boldsymbol{\eta }}}_{{\bf{t}}{\bf{o}}{\bf{t}},{\boldsymbol{\Delta }}{{\boldsymbol{\tau }}}_{{\boldsymbol{c}}}}$ (%)	Delivered Power to node (*μ*W)
single rectenna	0.04	−22.4	0.098	—	—	—	—	—	—
	0.39	−12.5	0.35	1227	0.735	9	0.59	23.34	160.7
	2.25	−4.9	0.925	—	—	—	—	—	—
rectenna-array	0.04	−19.4	0.201	—	—	—	—	—	—
	0.1237	−14.5	0.37	883	0.685	22.5	1.3	14.8	172
	2.25	−1.9	1.85	—	—	—	—	—	—

From a practical aspect of view, the single rectenna for −4.9 dBm RF power input presents 36.6% efficiency, and thus, the rectenna directly delivers to the optimum load dc power of 118 *μ*W at 0.51 V (Fig. 11). This means that the harvester is able to continuously supply a small electrical device such as a digital thermometer or a smoke detector with power consumption of 20 and 57 *μ*W, respectively^38^.

In this work, it will be tested the ability of the proposed harvester in supplying with power the backscatter sensor node presented in^29^. The latter has power consumption of the order of 100 *μ*W and 1.6 V voltage operation threshold. Based on Fig. 12, the single and the rectenna-array presents an open-circuit dc output voltage greater than 1.6 V when the power density is higher than 6.1 and 1.724 *μ*W/cm^2^, respectively. Hence, theoretically, it is possible the rectenna or rectenna-array to be directly connected with the sensor node and continuously supply the latter, without the use of any boost converter. However, the goal of this work is, among others, the design of a high sensitive harvester, and thus the latter should deliver dc power equal or higher than 100 *μ*W with voltage threshold of 1.6 V^29^ for low power density, equal or lower than 1 *μ*W. For the above reasons, the use of a boost converter is necessary.

The low power boost converter “bq25504” from Texas Instruments was used in this work^39^. The latter is an integrated circuit and performs power management and maximum power point tracking (MPPT) technique. Specifically, it has ultra-low quiescent current lower than 330 nA, cold start voltage of 330 mV, and once started, is able to harvest RF energy from voltage sources, greater than 80 mV. MPPT technique ensures the maximum extraction and transfer of energy from the source (e.g., rectenna) to the load (e.g., backscatter sensor node), by adjusting the input impedance of the boost converter^39^.

**Figure 13:**
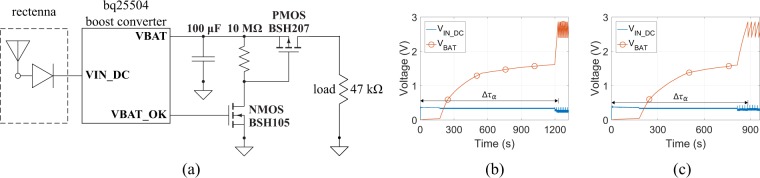
The total-harvester (i.e., rectenna(s) and the boost converter) schematic (**a**), the measured voltage dc across the rectenna output (${{\rm{V}}}_{\text{IN}\_\text{DC}}$) and the boost converter output (${{\rm{V}}}_{{\rm{BAT}}}$) for power density of 0.39 (**b**) and 0.1237 (**c**) *μ*W/cm^2^.

In order to estimate the end-to-end performance of the proposed harvesting system in a realistic scenario, the rectenna is connected through the boost converter circuit with the load (the schematic is depicted in Fig. 13a: it is similar to this one presented in^22^) and the rectenna’s voltage output (V_IN_DC_) and the voltage across the capacitor (${{\rm{V}}}_{{\rm{BAT}}}$) was measured via the data acquisition “DAQ NI USB-6356” instrument. The configuration of the commercial evaluation board of the “bq25504”^39^ was used. Specifically, once the voltage of the rectenna ${V}_{\mathrm{IN}\_\mathrm{DC}} > 330$ mV, cold start mode starts and energy flows through the boost converter to the 100 *μ*F capacitor, which is initially uncharged (i.e., ${{\rm{V}}}_{{\rm{BAT}}}=0$ mV at the beginning). The ${{\rm{V}}}_{\mathrm{BAT}\_\mathrm{OK}}$ pin produces a digital signal when the ${{\rm{V}}}_{{\rm{BAT}}}$ reaches 2.85 V and switches off when ${{\rm{V}}}_{{\rm{BAT}}}$ drops back to 2.4 V. The PMOS was used in combination with the bq25504 as a switcher since isolates the load from the supply system until the capacitor will be charged, in order to reduce the energy leakage. Analytically, as ${{\rm{V}}}_{{\rm{BAT}}} < 2.85$ V, ${{\rm{V}}}_{\mathrm{BAT}\_\mathrm{OK}}=0$ V (i.e., pin switched off), the PMOS “BSH207” stays off and thus, no energy flows to the load (all energy flows to capacitor at this time). But when ${{\rm{V}}}_{{\rm{BAT}}}=2.85$ V and until it drops back to 2.4 V, ${{\rm{V}}}_{\mathrm{BAT}\_\mathrm{OK}}\ne 0$, pin switches on and the inverted through the open drain NMOS “BSH105” signal drives the PMOS: now the latter turns on and current flows from the capacitor to the load. Next, when ${{\rm{V}}}_{{\rm{BAT}}}=2.4$ V, PMOS again turns off and capacitor is charging again until ${{\rm{V}}}_{{\rm{BAT}}}=2.85$ V: the above procedure is periodically repeated. For the sake of generality, a resistor of 47 k${\rm{\Omega }}$ was used as load in this work: the resistance is not arbitrary, since it was chosen in order to result in more than 100 *μ*W for voltage threshold higher than 2.4 V. All the above are in accordance with the goal of this work, which is to supply the backscatter sensor node in^29^, with power consumption of 100 *μ*W at 1.6 V. The choice of wide time period of RF harvesting and short time period of operation was the way was used in order to supply the backscatter sensor node^29^ with enough power to operate in a low power density environment^22^ as it will explained below, and thus, the *PMOS-switcher* was necessary in order to enhance the end-to-end efficiency by reducing the energy leakage.

Two scenarios were tested: first the harvester was the single rectenna and next the rectenna array. In each case, the sensitivity and the total (i.e., *end-to-end*) efficiency of the total-harvester (i.e., rectenna(s), boost converter and load) was investigated. The latter is given by,

7 $${\eta }_{{\rm{tot}}}=\frac{{W}_{{\rm{c}}}}{{W}_{{\rm{in}}}},$$

where, the *W*_c_ is the output dc energy, delivered to the load, as follows,

8 $${W}_{{\rm{c}}}=\frac{1}{2}\,C|{V}_{b}^{2}-{V}_{a}^{2}|$$

and *V*_*b*_, *V*_*a*_ denotes the high and low voltage across the capacitor, respectively, while,

9 $${W}_{{\rm{in}}}={\int }_{0}^{{\rm{\Delta }}\tau }\,{P}_{{\rm{in}}}{\rm{d}}t$$

is the input energy to the total-harvester within the time slot ${\rm{\Delta }}\tau$, where the capacitor is discharged (charged) from *V*_*b*_ to *V*_*a*_ (V_a_ to V_b_). It is noted that the latter efficiency takes into account all the sub-efficiencies, i.e., the antenna RE, the rectifier and the boost-converter efficiency and any other possible losses.

In this work, several measurements took place in order to find the total harvester sensitivity: for the single rectenna, sensitivity is $S=0.39$ *μ*W/cm^2^, which corresponds to ${P}_{{\rm{in}}}=-\,12.5$ dBm power input and to ${V}_{{\rm{o}}{\rm{.c}}{\rm{.}}}=0.35$ V open-circuit voltage, while for the rectenna-array, sensitivity is $S=0.1237$ *μ*W/cm^2^, ${P}_{{\rm{in}}}=-\,14.5$ dBm (via Eq. (13) for ${P}_{{\rm{cal}}}=-\,17.5$ dBm and *G* = 4.8 dB) and ${V}_{{\rm{o}}{\rm{.c}}{\rm{.}}}=0.37$ V (Table 3).

**Figure 14:**
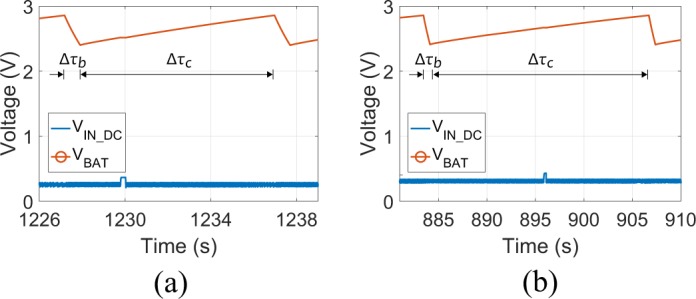
The measured voltage dc across the rectenna output (${{\rm{V}}}_{\text{IN}\_\text{DC}}$) and the boost converter output (${{\rm{V}}}_{{\rm{BAT}}}$) for power density of 0.39 (**a**) and 0.1237 (**b**) *μ*W/cm^2^: zoom-in of the Fig. 13b,c, respectively, after the cold start period.

Figures 13b,c and 14 depicts the measurement results using the “DAQ NI USB-6356” instrument: the boost converter’s dc voltage input (${{\rm{V}}}_{\text{IN}\_\text{DC}}$) and output (${{\rm{V}}}_{{\rm{BAT}}}$) for the single rectenna and the rectenna-array, when the power density is $0.39$ and $0.1237$ *μ*W/cm^2^, respectively, is presented. It is noted that, ${{\rm{V}}}_{\text{IN}\_\text{DC}}$ also represents the rectenna’s output, while ${{\rm{V}}}_{{\rm{BAT}}}$ also represents the dc voltage across the load, since the voltage drop across the $10$ M${\rm{\Omega }}$ is insignificant. In both cases, three distinct time periods should be considered. First, the *cold start* period (${\rm{\Delta }}{\tau }_{a}$), in which ${{\rm{V}}}_{\text{IN}\_\text{DC}}$ goes from $0$ to $2.85$ V. Second, the *discharging* period (${\rm{\Delta }}{\tau }_{b}$), when ${{\rm{V}}}_{\text{IN}\_\text{DC}}$ goes from $2.8$ to $2.4$ V, and third, the *charging* period (${\rm{\Delta }}{\tau }_{c}$), when ${{\rm{V}}}_{\text{IN}\_\text{DC}}$ changes again from $2.4$ to $2.85$ V. The last two operations, are periodically repeated.

For the single rectenna (Figs 13b and 14a), where $S=0.39$ *μ*W/cm^2^ (${P}_{{\rm{in}}}=-\,12.5$ dBm), the three latter periods are $1227$, $0.735$ and $9$ s. Thus, for the first period (${\rm{\Delta }}{\tau }_{a}=1227$ s) it is ${W}_{{\rm{c}}}=0.4061$ mJ and ${\eta }_{{\rm{tot}}}=0.59 \%$: the end-to-end efficiency is low during the cold-start period, since no external power was used, however, this period took place only once, at the beginning. For the third period (${\rm{\Delta }}{\tau }_{c}=9$ s) it is ${W}_{{\rm{c}}}=0.1181$ mJ and ${\eta }_{{\rm{tot}}}=23.34 \%$. Interest presents the second period: here, again ${W}_{{\rm{c}}}=0.1181$ mJ, however, because ${\rm{\Delta }}{\tau }_{b}=0.735$ s is very short, it is possible to estimate the delivered power to the load as ${W}_{{\rm{c}}}/{\rm{\Delta }}{\tau }_{a}=160.7$ *μ*W, and thus, the presented system is able to deliver more than 160 *μ*W every $9$ s, which is sufficient for the supply of the sensor tag, presented in^29^.

For the rectenna-array (Figs 13c and 14b), where $S=0.1237$ *μ*W/cm^2^ (${P}_{{\rm{in}}}=-\,14.5$ dBm), it is ${\rm{\Delta }}{\tau }_{a}=883$ s, ${\rm{\Delta }}{\tau }_{b}=0.685$ s and ${\rm{\Delta }}{\tau }_{c}=22.5$ s. Hence, for the *cold start* and the *charging* period it is again ${W}_{{\rm{c}}}=0.4061$ and $0.1181$ mJ, respectively, as expected, but now the end-to-end efficiency is $\mathrm{1.3 \% }$ and $\mathrm{14.8 \% }$, respectively. During the *discharging* period, the harvester system delivers to the load $0.1181$ mJ within $0.685$ s, or equivalently, the rectenna-array harvester delivers to the load more than $172$ *μ*W every 22.5 s. Table 3 summarizes all the above measurement results.

Finally, based on the latter measurement results and on^31,37^, the sensor tag presented in^29^ is able to be battery-less and to be supplied using entirely ambient RF energy, while, this is real, although no attempt has been made to optimize the performance of the boost dc-dc converter (was used with the initial, commercial evaluation board’s configurations^39^). It is noted also, that despite the measurements presented in^31,37^ are based on non-continuous signals, as cellular signals are, and thus, the ambient mentioned power levels are the average value, based on^40^, under certain load conditions non-continuous signals with time varying envelope may lead to higher RF-to-dc efficiency in comparison to continuous-wave signals. Additionally, in our case, since the proposed system is intended to supply the RF backscatter sensor node presented in^29^, and since the latter is used in a wireless sensor network, which adopts bi-static architecture, where an emitter illuminates the sensor with continuous signal at 868 MHz, the presented analysis responds to a realistic scenario.

## Discussion

This work presented the design and the measurement of an electrically small antenna, with omni-directional radiation pattern and high radiation efficiency, which is intrinsically matched to 50 ${\rm{\Omega }}$. It also presented the implementation and measurement of an electrically small rectenna with high RF-to-dc efficiency for low-power input and high sensitivity. In the rectenna, the antenna was directly impedance matched to the RF-to-dc rectifier, requiring no other matching network design. The harvester is able to supply continuously or through a boost converter battery-less small electrical devices, e.g. sensor-nodes, for extra low-power density levels: the latter was tested and shown through measurements. Based to our knowledge and according to the relevant literature, the electrically small rectenna presents better performance in terms of efficiency for low power input and sensitivity compared to prior-art designs: it is noted that the comparison, in order to be fair, relates to only electrically small antennas and rectennas Finally, future work will examine the use of the proposed rectenna in dense rectenna-arrays, with unit-cell size smaller enough than the wavelength.

## Methods

### Simulation setup

The antennas were simulated in terms of reflection coefficient, radiation pattern and current distribution via Ansys HFSS (ANSYS Inc., Canonsburg, PA, USA) with the Integral Equation (IE) method. For simulation for the rectifier the ADS software (Keysight Technologies) was used. Initially, full electromagnetic analysis with the MoM method was applied to the microstrip trace of the rectifier only, in order to estimate the fringing fields and the electromagnetic coupling between ports. Next, harmonic-balance was employed, taking into account the non-linear behaviour of the rectifier due to the diode. The latter, was modelled through its Spice model^41^.

### Measurement setup

**Figure 15:**
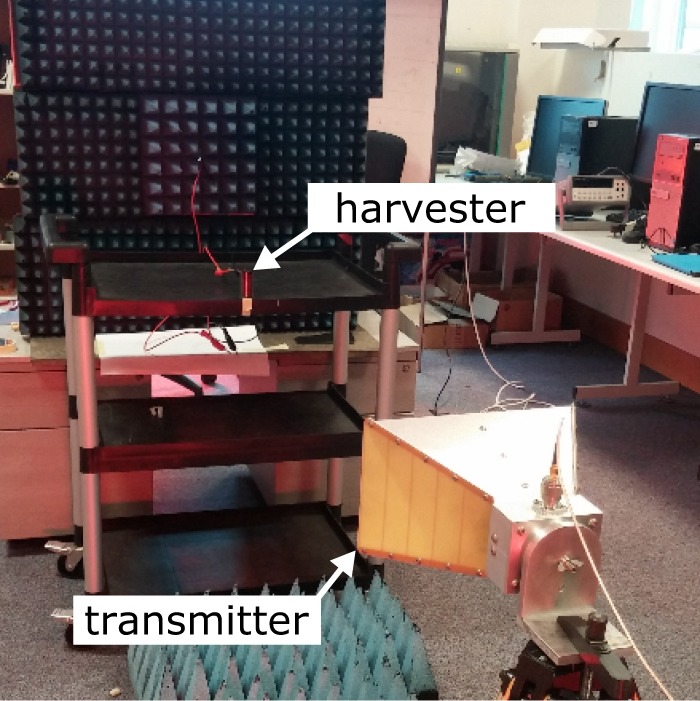
The measurement setup: rectenna (harvester) is placed in far-field region and the voltage across the load is measured.

The measurement setup is depicted in Fig. 15. A signal generator was connected to an antenna of known characteristics, which was placed at a specific point, transmitting power. A strip-SSRR antenna matched at 50 ${\rm{\Omega }}$ and with gain ${G}_{{\rm{cal}}}=1.8$ dB was also fabricated and placed at far-field distance away from the transmitter. This antenna, which hereafter will be called as *calibration antenna*, was connected to a spectrum analyser and the received power *P*_cal_ was measured. Hence, the power density is,

10 $$S=\frac{4\pi }{{\lambda }^{2}}\frac{{P}_{{\rm{cal}}}}{{G}_{{\rm{cal}}}}.$$

It is noted that the calibration antenna is co-polarized and co-aligned with the transmitter, while it has identical radiation pattern with the rectenna, since it has the same design with the antenna of the rectenna, but different impedance (metallic parts of the rectifier are too small compared with the antenna geometry, and thus, they do not affect the radiation pattern of the rectenna).

Next, the calibration antenna and the spectrum analyser were removed and the proposed rectenna was placed at exactly the same point. The dc voltage output across the load was measured. The rectenna RF-to-dc efficiency was estimated by,

11 $$\eta =\frac{{P}_{{\rm{out}}}}{{P}_{{\rm{in}}}}=\frac{{V}_{{\rm{R}}}^{2}/R}{S{A}_{{\rm{eff}}}},$$

where, ${P}_{{\rm{in}}}=S\,{A}_{{\rm{eff}}}$ and ${A}_{{\rm{eff}}}$ is the rectenna’s *effective area*^33^ which is given by,

12 $${A}_{{\rm{eff}}}=\frac{{\lambda }^{2}}{4\pi }G,$$

where *G* is the rectenna’s gain. Alternatively, based on Eqs (10–12) power input is given by,

13 $${P}_{{\rm{in}}}=\frac{G}{{G}_{{\rm{cal}}}}{P}_{{\rm{cal}}},$$

while the RF-to-dc efficiency is given by,

14 $$\eta =\frac{{P}_{{\rm{out}}}}{{P}_{{\rm{in}}}}=\frac{{V}_{{\rm{R}}}^{2}}{R}\frac{{G}_{{\rm{cal}}}}{G}\frac{1}{{P}_{{\rm{cal}}}}.$$

For the single rectenna at 868 MHz $G={G}_{{\rm{cal}}}=1.8$ dB in the horizontal plane (i.e., *xy*–plane), as explained, and thus ${P}_{{\rm{in}}}={P}_{{\rm{cal}}}$.
